# Supplementary material for: Prevalence and risk factors of computer vision syndrome—assessed in office workers by a validated questionnaire
Source: PeerJ. 2023 Mar 3;11:e14937. doi: 10.7717/peerj.14937 (PMC9987297; doi:10.7717/peerj.14937)
Supplement: Supplemental Information 2 [file peerj-11-14937-s002.docx]

**QUESTIONNAIRE OF SOCIODEMOGRAPHIC, OPTICAL CORRECTION AND EXPOSURE TO DIGITAL DEVICES VARIABLES**

- **SOCIODEMOGRAPHIC INFORMATION:**

Sex: Male 🞏 Female 🞏

Age: __________ years

Workplace:

University of Verona 🞏

Hospital Borgo Roma 🞏

Profession: ________________________

Department or operational unit: _______________________

- **GENERAL HEALTH HISTORY:**

1. **Do you take any medication?** YES 🞏 NO 🞏

**If yes, please list type and reason for its use:**

___________________________________________________ _________

- - **OCULAR HEALTH HISTORY:**

1. **Do you suffer or have you suffered from any of the following ocular disorders?**

Lazy eye (amblyopia) 🞏 Cataract 🞏 Strabismus (squint) 🞏

Conjunctivitis 🞏 Glaucoma 🞏 Eye injury 🞏

Keratoconus 🞏 Dry eye 🞏 Retinal problem 🞏

Other ________________

**If you have selected any of the above, please give details of when you had the ocular problem and if it is still present:** _ _____ ____

1. **Have you had any type of ocular surgery?** YES 🞏 NO 🞏

**Which?** Refractive surgery 🞏 Cataract surgery 🞏 Other __________________

1. **Are you currently undergoing any ocular pharmacological treatment?** YES 🞏 NO 🞏

**Which?** Ointments 🞏 Eye drops 🞏 Occasional artificial tears 🞏

Daily artificial tears 🞏 Other _______________

- - **USE OF CURRENT OPTICAL CORRECTION**

1. **In your day-to-day life, indicate the correct option:**

I do not wear glasses or contact lenses 🞏

I wear glasses:

Occasionally 🞏

For what activities? _________________________________________________

Almost always or always 🞏

I wear contact lenses:

No 🞏

Occasionally (sport, weekend…) 🞏

Almost always or always 🞏

I am under orthokeratology treatment 🞏

What refractive defect you have? Myopia 🞏 Hyperopia 🞏 Astigmatism 🞏

Presbyopia 🞏

1. **What do you use most frequently when working?**
2. Neither glasses nor contact lenses 🞏
3. Glasses 🞏
4. Contact lenses (if used at least 3 days/week) 🞏
5. **If you use glasses at work, what type are they?**

Monofocal 🞏 Bifocal 🞏

General progressive 🞏 Occupational progressive 🞏 Other _______________

- **EXPOSURE TO DIGITAL DEVICES INFORMATION**

1. **Please indicate the average number of hours per day you use digital devices for work (including all devices such as computer, laptop, tablet, smartphone...):**

_______________________________ hours/day

1. **For how long have you been using a computer or other digital device in the workplace? ___**_____ years
2. **When you use a computer or other digital devices at work, do you take regular scheduled breaks^a^ from time to time?** YES 🞏 NO 🞏

^a^ Intentional breaks to rest from the computer or other digital devices. This doesn’t include trips to the toilet, outings due to other activities that work time entails (e.g., meetings).

**Approximate duration of these breaks:** _______minutes

1. **Do you use air conditioning (hot/cold) in your workplace?**
2. Never
3. On rare occasions
4. Often
5. Always
6. **How many hours a day do you spend using a computer or other digital devices for non-work activities? (e-book, tablet, WhatsApp, mobile games...)**

______ hours/day

**SYMTOMS OF COMPUTER VISION SYNDROME**

The validated Italian version of the CVS-Q^©^ questionnaire, the CVS-Q IT^©^ questionnaire,^37^ was used to establish the symptomatology of the workers.

The CVS-Q IT^©^ is a scale that evaluates:

- **Frequency:**
  - Never: symptom does not occur at all
  - Occasionally: sporadic episodes or once a week
  - Often or always: 2 or 3 times a week to almost every day
- **Intensity**:
  - Moderate
  - Intense

of the following **16 ocular and visual symptoms related to the digital devices use**: burning, itching, feeling of a foreign body, tearing, excessive blinking, eye redness, eye pain, heavy eyelids, dryness, blurred vision, double vision, difficulty focusing for near vision, increased sensitivity to light, coloured halos around objects, feeling that sight is worsening and headache.

The questionnaire instructions ask the worker to indicate whether he/she experiences any of the following symptoms during the time he/she uses the computer at work. Subsequently the frequency and intensity data are recoded to calculate the severity of each symptom using the following mathematical expression:

$$\mathrm{Score}= \sum_{i=1}^{16} (\mathrm{frequency} \mathrm{of} \mathrm{symptom}{occurrence)}_{i}\times{(intensity of symptom)}_{i}$$

At the end, a total score is obtained. If the **total score is ≥ 7,** the subject suffers computer vision syndrome.

**OCULAR SURFACE AND TEAR OPHTHALMIC TESTS**

1. **Break-up time (BUT)**

Right eye: __________ seconds

Left eye: ___________ seconds

1. **Corneal staining** (scale CCLRU: Cornea and Contact Lens Research Unit)

**
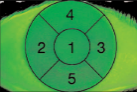
**Right eye:

- Area
- Number of staining points: __________

**
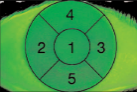
**Left eye:

- Area
- Number of staining points: __________

1. **Schirmer II**

Right eye: __________ millimetres

Left eye: ___________ millimetres
