# Supplementary material for: Prevalence and risk factors of computer vision syndrome—assessed in office workers by a validated questionnaire
Source: PeerJ. 2023 Mar 3;11:e14937. doi: 10.7717/peerj.14937 (PMC9987297; doi:10.7717/peerj.14937)
Supplement: Supplemental Information 3 [file peerj-11-14937-s003.docx]

| **Supplemental file.** Association between computer vision syndrome and sociodemographic, ocular health, optical correction variables, and exposure to digital devices: crude odds ratio (cOR) with 95% confidence intervals (95% CI) | | |
| --- | --- | --- |
|  | **cOR** | **95% CI** |
| **Sex** |  |  |
| Male | 1 |  |
| Female | 3.422 | 1.938 – 6.044 |
| **Age (years)** |  |  |
| ≤ 40 | 1 |  |
| > 40 | 1.295 | 0.730 – 2.297 |
| **Workplace** |  |  |
| University of Verona | 1 |  |
| Hospital Borgo Roma | 1.163 | 0.568 – 2.379 |
| **General pharmacological treatment** |  |  |
| No | 1 |  |
| Yes | 1.176 | 0.668 – 2.070 |
| **Past ocular disorders** |  |  |
| No | 1 |  |
| Yes | 0.740 | 0.305 – 1.794 |
| **Ocular surgery** |  |  |
| No | 1 |  |
| Yes | 0.725 | 0.199 – 2.649 |
| **Ocular pharmacological treatment** |  |  |
| No | 1 |  |
| Yes | - | - |
| **Regular optical correction** |  |  |
| No | 1 |  |
| Yes | 3.130 | 1.646 – 5.952 |
| **Use of glasses to work** |  |  |
| No | 1 |  |
| Yes | 3.108 | 1.726 – 5.596 |
| **Lens design at work^†^** |  |  |
| Nothing | 1 |  |
| Monofocal distance | 3.712 | 1.830 – 7.532 |
| Monofocal near | 2.121 | 0.971 – 4.633 |
| General progressive | 3.182 | 1.196 – 8.467 |
| Occupational progressive | 4.242 | 1.098 – 10.069 |
| **Presbyopia** |  |  |
| No | 1 |  |
| Yes | 1.177 | 0.682 – 2.030 |
| **Occupational use of digital devices (hours/day)** |  |  |
| ≤ 6 | 1 |  |
| > 6 | 2.204 | 1.203 – 4.038 |
| **Years working with digital devices** |  |  |
| ≤ 10 | 1 |  |
| 11 – 20 | 1.324 | 0.693 – 2.532 |
| > 20 | 1.364 | 0.682 – 2.725 |
| **Scheduled breaks during work with digital devices** |  |  |
| No | 1 |  |
| Sí | 0.827 | 0.374 – 1.828 |
| **Duration of breaks (minutes)** |  |  |
| ≤ 5 | 1 |  |
| 6 – 10 | 1.117 | 0.601 – 2.075 |
| > 10 | 0.895 | 0.437 – 1.832 |
| **Use of air conditioning at work** |  |  |
| Never or rarely | 1 |  |
| Often or always | 1.794 | 0.871 – 3.693 |
| **Use of digital devices for leisure (hours/day)** |  |  |
| ≤ 2 | 1 |  |
| > 2 | 1.058 | 0.534 – 2.097 |
| **Total use of digital devices (hours/day)** |  |  |
| ≤ 6 | 1 |  |
| 6 – 8 | 1.504 | 0.787 – 2.873 |
| > 8 | 2.189 | 1.057 – 4.536 |
| -: for this variable the association cannot be calculated.  **^†^** The category “bifocal” of these variable has been excluded from the analysis of the association since there was only one person. | | |
